# Novel Mutation Hotspots within Non-Coding Regulatory Regions of the Chronic Lymphocytic Leukemia Genome

**DOI:** 10.1038/s41598-020-59243-5

**Published:** 2020-02-12

**Authors:** Adrián Mosquera Orgueira, Beatriz Rodríguez Antelo, José Ángel Díaz Arias, Nicolás Díaz Varela, Natalia Alonso Vence, Marta Sonia González Pérez, José Luis Bello López

**Affiliations:** 1 Health Research Institute of Santiago de Compostela (IDIS), Santiago de Compostela, Spain; 2 Complexo Hospitalario Universitario de Santiago de Compostela (CHUS), Division of Hematology, SERGAS, Santiago de Compostela, Spain; 3 University of Santiago de Compostela, Santiago de Compostela, Spain

**Keywords:** Molecular medicine, Chronic lymphocytic leukaemia

## Abstract

Mutations in non-coding DNA regions are increasingly recognized as cancer drivers. These mutations can modify gene expression in *cis* or by inducing high-order chormatin structure modifications with long-range effects. Previous analysis reported the detection of recurrent and functional non-coding DNA mutations in the chronic lymphocytic leukemia (CLL) genome, such as those in the 3′ untranslated region of *NOTCH1* and in the *PAX5* super-enhancer. In this report, we used whole genome sequencing data produced by the *International Cancer Genome Consortium* in order to analyze regions with previously reported regulatory activity. This approach enabled the identification of numerous recurrently mutated regions that were frequently positioned in the proximity of genes involved in immune and oncogenic pathways. By correlating these mutations with expression of their nearest genes, we detected significant transcriptional changes in genes such as *PHF2* and *S1PR2*. More research is needed to clarify the function of these mutations in CLL, particularly those found in intergenic regions.

## Introduction

A major part of mutations in the cancer genome occur in non-coding DNA regions, and their function is still beginning to be understood^1^. Non-coding DNA comprises approximately 98% of the human genome, but recent research has proven that most of these regions are either part of regulatory motifs or actively transcribed to RNA^2,3^. These mutations can induce functional genomic changes by altering the binding of transcription factors or by inducing high-order chromatin structural modifications^2,4^. For example, mutations in 5′ and 3′ untranslated regions (UTRs) may disturb RNA structural conformation, modify microRNA binding sites or disrupt polyadenylation signals^2^. In a similar fashion, mutations affecting non-protein coding genes such as microRNA and long intergenic RNA genes (lincRNAs) are known cancer driver events^2,5^. Different studies have evidenced that the expression of genes such as *BRCA1, CDH10, CCND1, MALAT1, PAX5*, *RB1, SDHD*, *TERT*, *TOX3*, and *TAL1* is influenced by non-coding DNA mutations in regulatory regions of the cancer genome^1,6,7^. The *Pancancer Analysis of Whole Genomes* (PCAWG) project has revealed the existence of common and tumor-specific recurrently mutated functional elements near known cancer drivers^7^. Some of these driver mutations can induce long-range changes in genome organization and trigger abnormal expression of distant oncogenes and tumor suppressors^8^. Furthermore, the sequence distribution of these driver mutations is not random. Hornshøj *et al*. (2018) identified a significant enrichment in conserved CCCT-binding factor (CTCF) binding sites among recurrently mutated non-coding DNA regions with cancer specificity^6^. Similarly, Line *et al*. (2019) identified 21 recurrently altered CTCF-rich insulator regions in the cancer genome, and elegantly demonstrated that some of these mutations drive tumor proliferation^9^.

Chronic Lymphocytic Leukemia (CLL) is among the most frequent lymphoproliferative disorders, and it is characterized by its remarkable clinical heterogeneity. Recent efforts by Puente *et al*.^10^ enabled the discovery of 24 recurrently mutated non-coding genomic regions in the CLL genome, some of which are associated with functional changes such as mutations in the 3′UTR of *NOTCH1* and in the *PAX5* super-enhancer. Nevertheless, both the sparsity of annotations in non-coding DNA regions and the difficult functional classification of non-coding DNA mutations hinder a better understanding of the non-coding cancer genome, which probably harbors multiple deregulated elements yet to discover. In this analysis, we analyzed whole genome sequencing (WGS) data using a best-practice mutation detection pipeline. Then, we identified signals of positive selection of mutations in regulatory regions. Finally, our last attempt was to analyze if any of these recurrent mutations in non-coding DNA regions were associated with abnormal expression of the nearest gene. Our results point toward the existence of dozens of mutation-enriched regulatory regions near cancer and immune-related genes, some of which influence local gene expression.

## Methods

### Data origin

Whole genome sequencing files produced by the *International Cancer Genome Consortium*^11^ were obtained from the *European Genome-Phenome Archive* under accession code *EGAD00001001466*. Gene expression from microarray data of the same set of patients was obtained from *EGAD00010000875*.

### Data analysis

130 tumor-normal matched CLL whole genomes were processed using the bcbio-nextgen pipeline, which provides best practices for analyzing high throughput sequencing data^12^. Low complexity regions, areas with abnormally high coverage, sequences with single nucleotide stretches >50 bp and loci with alternative or unplaced contigs in the reference genome were not analyzed. Some polymorphic regions are prone to be classified as highly mutated due to artifacts or biases in the sequencing process, and suspicious elements were manually removed from downstream analysis. Single nucleotide and indel mutation detection was performed with *vardict*^13^, *varscan*^14^, *mutect2*^15^ and *freebayes*^16^ using default bcbio-nextgen parameters. Only variants with a minimum sequencing depth (DP) of 10 and a genotype quality (GQ) above 20 Phred in both tumor and normal samples were analyzed. A mutation was reported when detected by at least two different mutation callers. Mutations were annotated to the 1000G^17^, gnomAD^18^ and ExAc^19^ databases in order to filter likely germline variants. All mutations with a minimum allele frequency >0.001 in any population were discarded from the analysis.

### Region annotation

Annotations corresponding to promoter regions, 5′UTR, 3′UTR and lincRNAs were retrieved from Genecode version 18^20^. DNAse hypersensitivity (DHS) regions and Transcription Factor Binding Sites (TFBS) tracks from the ENCODE^21^ project were obtained from Lochovsky *et al*.^22^. Similarly, we used enhancer regions from the GeneHancer database^23^, and analyzed those that were supported by two or more sources of evidence (*“elite” enhancers*). Regulatory regions within telomeric and centromeric positions were discarded.

Two different methods were used to identify areas with evidence of positive selection of mutations: *LARVA*^22^ and *OncodriveFML*^24^. *LARVA* models the mutation counts of each target region as a β-binomial distribution in order to handle overdispersion. Furthermore, *LARVA* also includes replication timing information in order to estimate local mutation rate, and provides a β-binomial distribution adjusted for replication timing which is used to compute p-values. On the other hand, *OncodriveFML* is designed to analyze the pattern of somatic mutations across tumors in both coding and non-coding genomic regions. *OncodriveFML* uses functional predictions in order to identify signals of positive selection. *OncodriveFML* was run with CADD v1.3 scores and default parameters. TFBS tracks were not analyzed with OncodriveFML due to high computational demands. Regions were labeled as significantly mutated if the q-value was <0.05 with any of the two methods.

### Gene expression analysis and association with recurrent non-coding DNA mutations

Background correction, normalization and log2-transformation of microarray gene expression data was performed with the RMA algorithm^25^. In the case of genes targeted by multiple probes, the median expression was calculated. The Wilcoxon-Rank sum test was used to detect changes in gene expression between mutated and wild-type cases. Non-coding regulatory genomic regions cannot be directly ascribed to any gene, and they can affect the transcription of virtually any part of the genome. However, this study is underpowered to detect long-range interactions due to small sample size and the need of extreme p-values passing multiple-testing correction. Therefore, we centered our efforts on changes in expression of the nearest gene. We annotated the closest gene to each recurrently mutated non-coding genomic region as the nearest transcription start site to the middle position of the corresponding region. In the case of multiple overlapping regulatory regions, we selected the most significant one for downstream analysis. P-values were adjusted for multiple testing using the FDR method, with a significance threshold of 0.05.

## Results

### Mutation distribution

397,433 non-coding DNA mutations were detected in the genome of this CLL cohort. Most of these were either intergenic (45.46%) or intronic (42.12%). The remaining mutations were located in 5′ flanks (5.83%), 3′ flanks (5.30%), RNA genes (0.64%), 3′UTRs (0.52%) and 5′UTRS (0.13%). Most of the mutations were single nucleotide variants (92.96%), whereas 4.57% and 2.47% were short deletions and insertions, respectively.

### Regions significantly enriched in mutations

**Figure 1:**
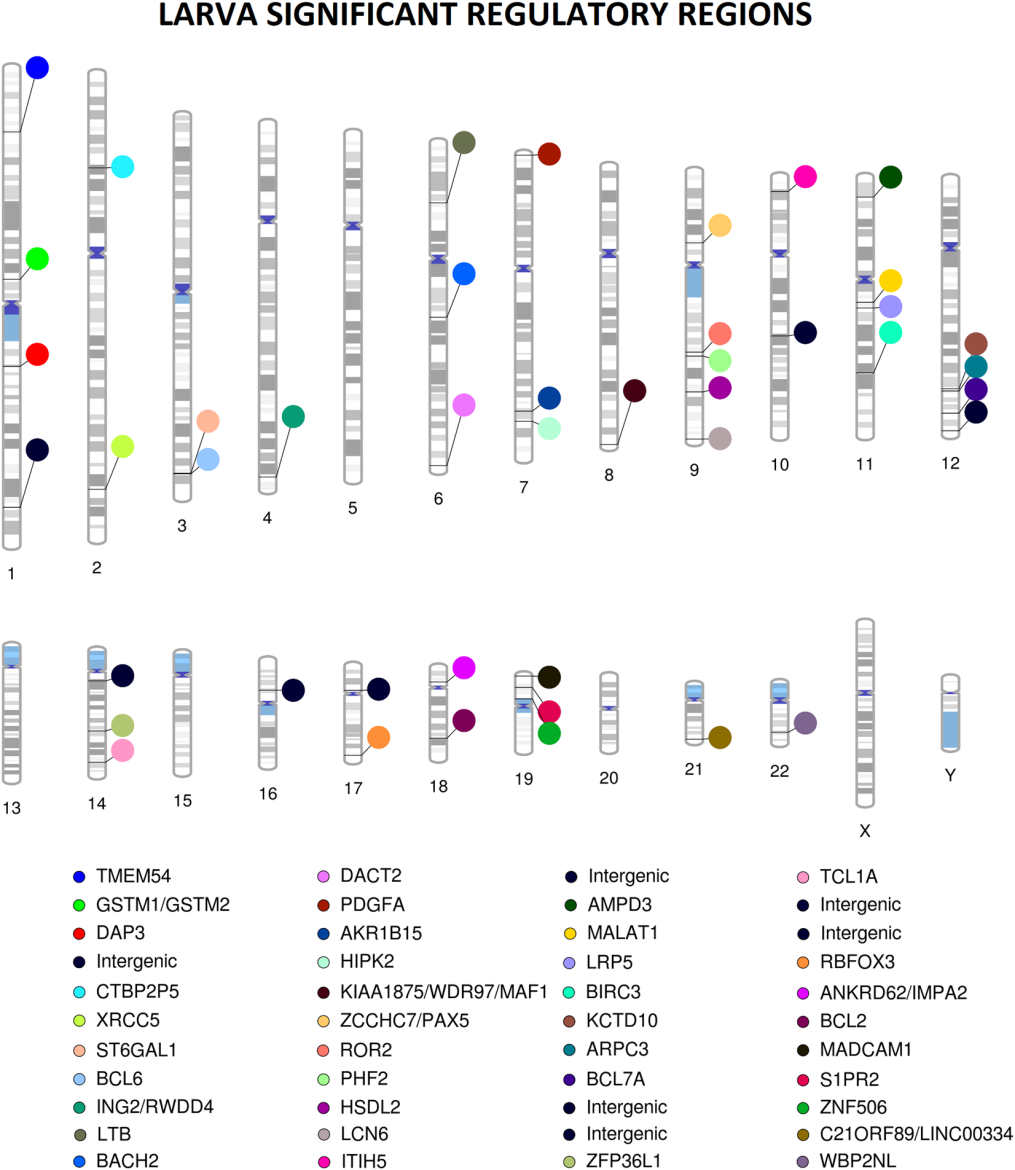
Chromosomal ideogram representing the different gene affected by recurrent non-coding mutations according to *LARVA*.

*LARVA* detected significant mutation enrichments (q-value < 0.05) in 120 TFBS, 16 DHS regions, 10 enhancers, 4 promoters, 2 5′UTRs and 1 lincRNA (Table 1, Supplementary Tables 1–6). No relevant inflation in p-value distribution was observed. (Supplementary Fig. 1). These regions were located in 44 different genomic loci (Fig. 1). The most recurrently mutated promoters were those of *TCL1A* (q-value 3.32 × 10^−4^), *LCN6* (q-value 4.17 × 10^−3^), *ZFP36L1* (q-value 3.25 × 10^−2^) and *WDR97* (q-value 0.04); and the most significantly mutated enhancers were *GH01J229147* (intergenic region chr1:229283343–229284982, q-value 5.79 × 10^−6^) and *GH07J000467* (*PDGFA* gene, q-value 8.53 × 10^−4^). The DHS regions chr4:184474905–184475055 (*ING2*/*RWDD4* locus, q-value 1.42 × 10^−5^), chr21:46673965–46674115 (*C21ORF89*/*LINC00334* locus, q-value 1.38 × 10^−4^), chr14:96179960–96180110 (*TCL1A* locus, q-value 3.98 × 10^−4^) and chr9:115161245–115161395 (*HSDL2* locus, q-value 3.98 × 10^−4^) were the most recurrently mutated among their class (Supplementary Table 2). Furthermore, up to 120 significantly mutated TFBS regions were detected, affecting 19 different genes and 3 intergenic regions. The most recurrently mutated regions were located in chr1:155666495–155666977 (*DAP3* gene, q-value 3.14 × 10^−10^), chr14:96179816–96180607 (*TCL1A* gene, 1.38 × 10^−4^), chr3:186782686–186783907 (*BCL6* gene, 3.52 × 10^−4^), chr7:507220–508145 (*PDGFA* gene, 8.15 × 10^−4^) and chr18:12086057–12086469 (*ANKRD62* gene, 8.30 × 10^−4^) (Supplementary Table 4).

**Table 1:** Summary of the regions most significantly enriched in mutations according to *LARVA*.

Chromosome	Start	Stop	Mutation count	p-value (bbd)	FDR	Gene	SHM target	Type of Regulator
chr1	155666495	155666977	16	2.20E-16	3.14E-10	*DAP3*	No	TFBS
chr1	229283343	229284982	28	1.58E-10	5.79E-06	Intergenic	No	ENHANCER
chr3	186782686	186783907	26	1.34E-09	3.52e-04	*BCL6*	Yes	TFBS
chr4	184474905	184475055	13	3.92E-11	1.42E-05	*ING2/RWDD4*	No	DHS
chr7	507064	509696	17	4.65E-08	8.53e-4	*PDGFA*	No	ENHANCER
chr7	507220	508145	17	3.85E-09	8.15E-4	*PDGFA*	No	TFBS
chr9	115161245	115161395	11	2.98E-09	3.98e-4	*HSDL2*	No	DHS
chr11	65265233	65273940	10	2.04E-08	4.50e-4	*MALAT1*	Yes	lincRNA
chr14	96179060	96180273	25	2.34E-08	3.32E-4	*TCL1A*	Yes	PROMOTER
chr14	96179721	96180690	22	1.36E-09	3.52E-4	*TCL1A*	Yes	TFBS
chr14	96179799	96180653	21	6.70E-10	2.25E-4	*TCL1A*	Yes	TFBS
chr14	96179816	96180607	21	2.67E-10	1.38E-4	*TCL1A*	Yes	TFBS
chr14	96179960	96180110	12	3.03E-09	3.98E-4	*TCL1A*	Yes	DHS
chr21	46673965	46674115	12	7.33E-10	1.38E-4	*C21ORF89*/*LINC00334*	No	DHS

Other significant enhancer regions were located in the proximity of genes involved in apoptosis (*BCL2* and *BIRC3*), cell cycle control (*WBP2NL*), cytoskeleton and extracellular matrix formation (*ARPC3* and *ITIH5*), gene expression regulation and chromatin remodelling (*BCL7A*, *PAX5* and *PHF2*), genome integrity (*XRCC5* and *ZNF506*), gene expression regulation (*MALAT1* and *RBFOX3*), intracellular signalling (*DACT2*, *HIPK2*, *IMPA2*, *KCTD10*, *ROR2* and *S1PR2*), immune pathways (*BACH2*, *LTB* and *MADCAM1*) and metabolism (*AKR1B15*, *AMPD3*, *GSTM1/GSTM2*, *LRP5* and *ST6GAL1*) (Supplementary Tables 1–6). Recurrent mutations were also found near less well-characterized genes such as *TMEM54* and *CTBP2P5*, as well as within intergenic regions such chr14:26068671–26069217 and chr1:229283491–229285693.

Finally, *OncodriveFML* identified 4 regions significantly enriched in likely functional mutations (Supplementary Tables 7 and 8). No relevant inflation in p-value distribution was observed (Supplementary Fig. 2). These regions were the enhancer *GH14J089855* (q-value 2.54 × 10^−3^) encoded within an intronic region of *EFCAB11*, two DHS regions in the proximity of *EGR* and *WBNPL2* (q-values 0.01 and 0.03, respectively), and one intergenic DHS region located in chr8:127155560–127155710 (q-value 1.22 × 10^−3^).

### Mutations associated with changes in gene expression

We studied the association of regions enriched in mutations with changes in the expression of their respective nearest genes. Although this type of analysis is limited by low sample size, we detected significant associations in some cases. We tested if patients with at least one mutation in these regulatory regions were accompanied by changes in expression of the nearest gene. Significant associations were observed in 3 genes, namely *PHF2* (q-value 0.02, 95% CI [−0.295, −0.048]), *RPL39L* (q-value 0.04, 95% CI [0.018, 0.217]) and *S1PR2* (q-value 0.03, 95% CI [0.033, 0.38]) (Supplementary Table 9).

## Discussion

Mutations in the non-coding part of the genome constitute the “dark-matter” of cancer genomics^2^. Growing evidence indicates that many of these mutations occur in conserved motifs and loci under epigenetic control, and some of these play fundamental roles in cancer biology and disease prognosis^1–3,6–9^. Using WGS data produced by the *ICGC*, we identified dozens of recurrently mutated regulatory regions in the CLL genome. Among these, 10 were previously reported by the original analysis performed by Puente *et al*.^10^, namely those near *BACH2*, *BLC2, BCL6*, *BCL7A*, *BIRC3, S1PR2, PCDH15*, *ZCCHC7*/*PAX5* and *ZFP36L1*. Numerous novel regions were also enriched in non-coding DNA mutations, including transcription factor binding sites, DNAse hypersensitivity regions, 5′UTR regions, promoters, enhancers and non-coding RNAs. These events were frequently found in the vicinity of genes previously vinculated with oncogenic pathways. Indeed, the most significantly mutated regions were a SETB1 binding site within the first intron of *DAP3*, a GTP-binding protein that participates in the apoptosis pathway^26^; and a DNAse hypersensitivity region downstream to *ING2*, a well-characterized tumor suppressor^27^. Other highly mutated regulatory regions affected cancer-related genes such as *DACT2*^28^, *ERG*^29^,_,_ *HIPK2*^30^, *ITIH5*^31^, *LRP5*^32^, *MAF1*^33^, *MALAT1*^34^, *PHF2*^35^, *PDGFA*^36^, *RBFOX3*^37^, *ROR2*^38^, *ST6GAL1*^39^ and *XRCC5*^40^; and others were detected near genes involved in immunity, such as *LTB*^41^ and *MADCAM1*^42^. Overall, only three of the novel genes (*LTB*, *MALAT1* and *ST6GAL1*) were previously defined as targets of somatic hypermutation in B cell lymphomas^43^. Finally, it is worthwhile to mention that recurrent and even highly significant enrichments were detected around barely characterized genes (e.g. *C21ORF89*/*LINC0334*) and intergenic regions.

The reported mutations can either be bystander or have functional implications related to their potential to modify gene expression or to induce high-order chromatin structural changes. Although limited by low sample size, we devised significant changes in the expression of *PHF2*, *S1PR2* and *RPL39L*. These three genes are involved in the regulation of important oncogenic processes. *PHF2* encodes a histone demethylase with tumor suppressor activity^35^. *S1PR2* participates in the TGF-β pathway and acts as a tumor suppressor of B cell lymphomas^44^. Finally, *RPL39L*^45^ is involved in cancer stem cell self-renewal and hypoxia response. These results are concordant with other reports of non-coding regulatory mutations driving gene expression changes in B-cell lymphomas^46–48^.

The combination of an optimized mutation detection pipeline with statistical tests specifically designed to handle non-coding DNA mutations has enabled the detection of novel putative regulatory driver regions in the CLL genome. These regions were mostly located in the vicinity of genes implicated in oncogenic and immune pathways, although several recurrently mutated intergenic regions were detected too. Furthermore, we could confirm the association of some of these events with altered expression of their respective genes. We expect that our results, along with those published by other groups, will promote an improved characterization of the non-coding mutational drivers of CLL.

## Supplementary information

Supplementary Figures 1 and 2.

Supplementary Table 1.

Supplementary Table 2.

Supplementary Table 3.

Supplementary Table 4.

Supplementary Table 5.

Supplementary Table 6.

Supplementary Table 7.

Supplementary Table 8.

Supplementary Table 9.

Supplementary Figure Legends.

## Data Availability

There is not data to deposit.
