# Supplementary figures and images for: Novel Mutation Hotspots within Non-Coding Regulatory Regions of the Chronic Lymphocytic Leukemia Genome
Source: Sci Rep. 2020 Feb 12;10:2407. doi: 10.1038/s41598-020-59243-5 (PMC7015923; doi:10.1038/s41598-020-59243-5)

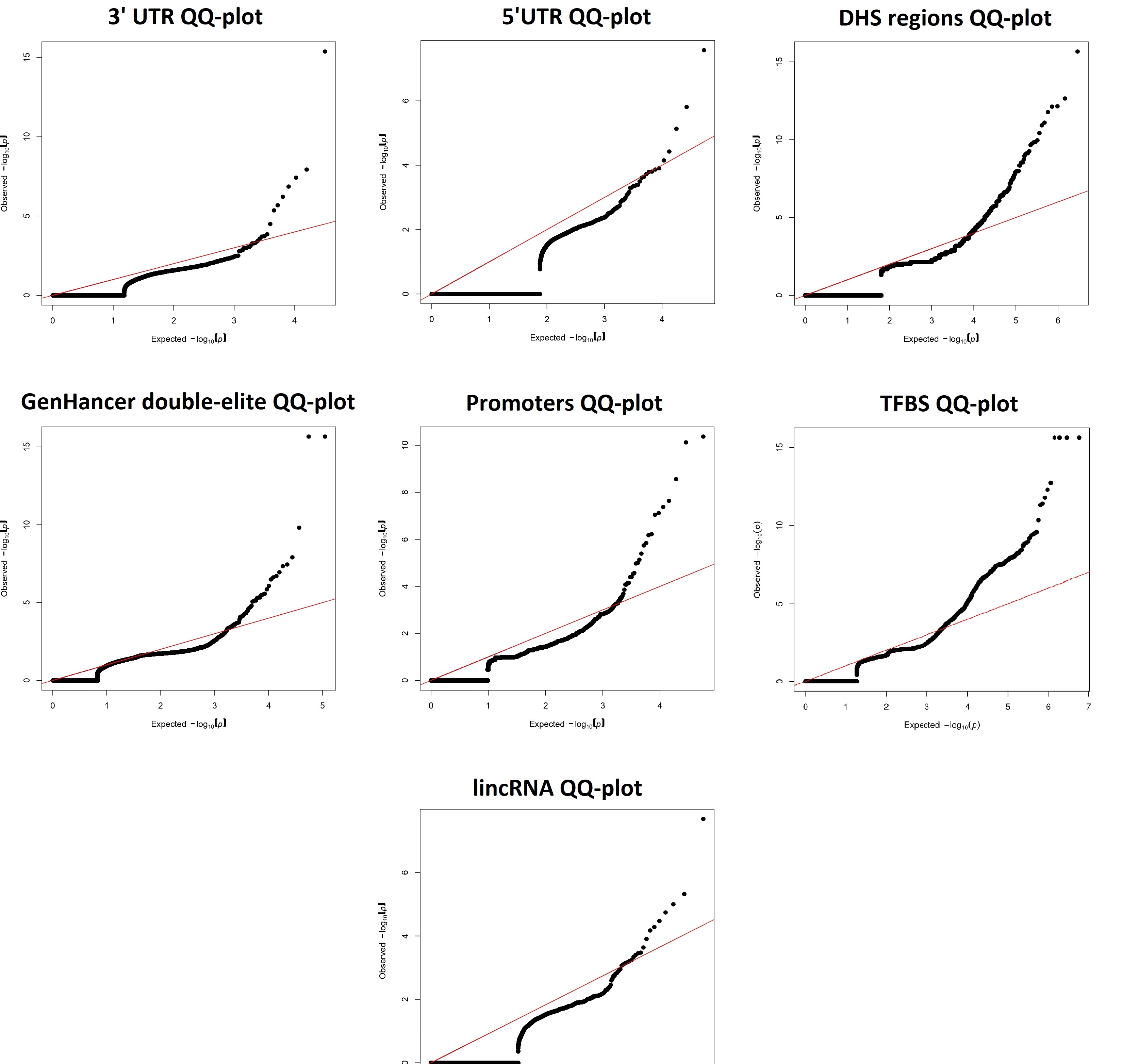

Supplement: Supplementary file 1 — Supplementary Figures 1 and 2. [file 41598_2020_59243_MOESM1_ESM.zip › Supplementary Figure 1.jpg]

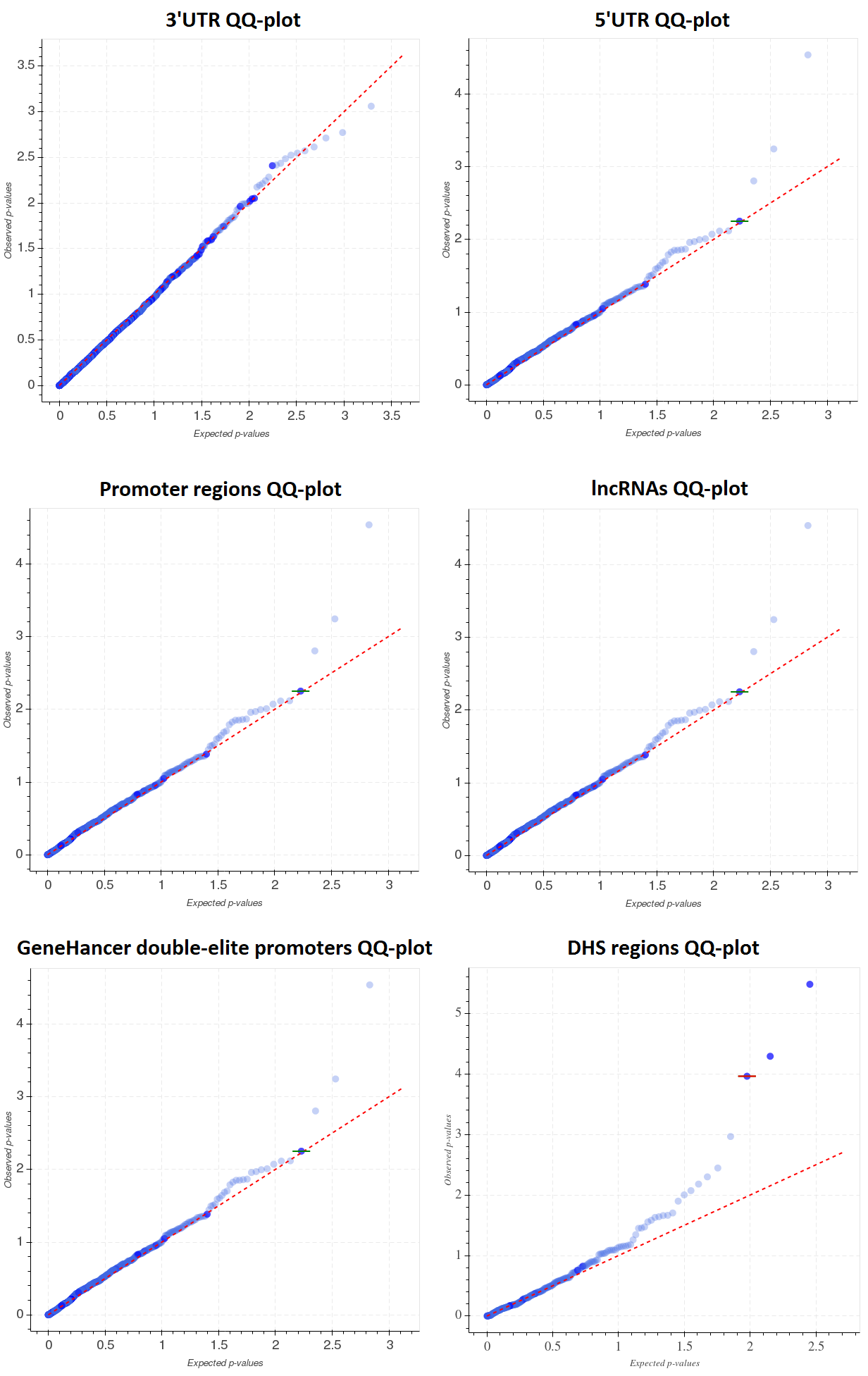

Supplement: Supplementary file 1 — Supplementary Figures 1 and 2. [file 41598_2020_59243_MOESM1_ESM.zip › Supplementary Figure 2 (1).png]
