# Supplementary material for: Novel Mutation Hotspots within Non-Coding Regulatory Regions of the Chronic Lymphocytic Leukemia Genome
Source: Sci Rep. 2020 Feb 12;10:2407. doi: 10.1038/s41598-020-59243-5 (PMC7015923; doi:10.1038/s41598-020-59243-5)
Supplement: Supplementary file 11 — Supplementary Figure Legends. [file 41598_2020_59243_MOESM11_ESM.docx]

**NOVEL MUTATION HOTSPOTS WITHIN NON-CODING REGULATORY REGIONS OF THE CHRONIC LYMPHOCYTIC LEUKEMIA GENOME**

**Running Title: Novel non-coding mutation hotspots in CLL**

**Authors**

Adrián Mosquera Orgueira,1,2,3 Beatriz Antelo Rodríguez,1,2,3 José Ángel Díaz Arias1,2, Nicolás Díaz Varela1,2, Natalia Alonso Vence1,2, Marta Sonia González Pérez1,2 and José Luis Bello López1,2,3

1Health Research Institute of Santiago de Compostela (IDIS); 2Complexo Hospitalario Universitario de Santiago de Compostela (CHUS), Division of Hematology, SERGAS; 3University of Santiago de Compostela.

**Corresponding author**

Adrián Mosquera Orgueira

Address: Hospital Clínico Universitario de Santiago de Compostela, Servicio de Hematología, planta 1, Avenida da Choupana s/n, Santiago de Compostela, 15706, Spain

E-mail: adrian.mosquera@live.com, Fax: 0034981950172

**Supplementary Figure 1.** Quantile-Quantile plots of LARVA-based p-values in the different regulatory regions analyzed.

**Supplementary Figure 2.** Quantile-Quantile plots of OncodriveFML-based p-values in the different regulatory regions analyzed. The genomic elements that have a lighter color in the plot are the ones for which the number of mutated samples does not reach the minimum required to perform the multiple test correction (2 samples). All the genomic regions above the red line in the plot represent those with a Q-value below 0.1. The ones between the green line and the red line are the ones with a Q-value between 0.25 and 0.1.
